# Clinical spectrum and long-term outcomes of mpox: a cohort study spanning from acute infection to six-month follow-up

**DOI:** 10.1186/s12879-024-09191-6

**Published:** 2024-03-15

**Authors:** Christoph Zeyen, Maria Kinberger, Helene Kriedemann, Frieder Pfäfflin, Pinkus Tober-Lau, Leu Huang, Victor Max Corman, Alexander Nast, Leif Erik Sander, Florian Kurth, Ricardo Niklas Werner

**Affiliations:** 1 Department of Dermatology, Venereology and Allergology, Division of Evidence-Based Medicine (dEBM), Charité– Universitätsmedizin Berlin, corporate member of Freie Universität Berlin and Humboldt-Universität zu Berlin, Charitéplatz 1, 10117 Berlin, Germany; 2 Department of Infectious Diseases, Respiratory Medicine and Critical Care, Charité– Universitätsmedizin Berlin, corporate member of Freie Universität Berlin and Humboldt-Universität zu Berlin, Campus Virchow Klinikum, Augustenburger Platz 1, 13353 Berlin, Germany; 3 Institute of Virology, Charité– Universitätsmedizin Berlin, corporate member of Freie Universität Berlin and Humboldt-Universität zu Berlin, Charitéplatz 1, 10117 Berlin, Germany

**Keywords:** Monkeypox, Mpox, Mpxv, Quality of life, Scar assessment, Sequelae

## Abstract

**Background:**

Cases of mpox have been reported worldwide since May 2022. Limited knowledge exists regarding the long-term course of this disease. To assess sequelae in terms of scarring and quality of life (QoL) in mpox patients 4–6 months after initial infection.

**Methods:**

Prospective observational study on clinical characteristics and symptoms of patients with polymerase chain reaction (PCR)-confirmed mpox, including both outpatients and inpatients. Follow-up visits were conducted at 4–6 months, assessing the Patient and Observer Scar Assessment Scale (POSAS), the Dermatology Life Quality Index (DLQI) and sexual impairment, using a numeric rating scale (NRS) from 0 to 10.

**Results:**

Forty-three patients, age range 19–64 years, 41 men (all identifying as MSM) and 2 women, were included. Upon diagnosis, skin or mucosal lesions were present in 93.0% of cases, with 73.3% reporting pain (median intensity: 8, Q1-Q3: 6–10). Anal involvement resulted in a significantly higher frequency of pain than genital lesions (RR: 3.60, 95%-CI: 1.48–8.74). Inpatient treatment due to pain, superinfection, abscess or other indications was required in 20 patients (46.5%). After 4–6 months, most patients did not have significant limitations, scars or pain. However, compared to patients without such complications, patients with superinfection or abscess during the acute phase had significantly more extensive scar formation (median PSAS: 24.0 vs. 11.0, *p* = 0.039) and experienced a significantly greater impairment of their QoL (median DLQI: 2.0 vs. 0.0, *p* = 0.036) and sexuality (median NRS: 5.0 vs. 0.0, *p* = 0.017).

**Conclusion:**

We observed a wide range of clinical mpox manifestations, with some patients experiencing significant pain and requiring hospitalization. After 4–6 months, most patients recovered without significant sequelae, but those with abscesses or superinfections during the initial infection experienced a significant reduction in QoL and sexuality. Adequate treatment, including antiseptic and antibiotic therapy during the acute phase, may help prevent such complications, and hence, improve long-term outcomes.

## Background

Since its identification in humans in 1970, the mpox virus (MPXV) has been known to cause a disease similar to smallpox, mainly in Central and West Africa [1]. However, since May 2022, mpox (formerly termed monkeypox) has emerged outside of its former endemic areas, leading to outbreaks with sustained chains of anthropogenic transmission in new regions. As of July 2023, more than 88,000 cases and 147 deaths have been reported in 112 countries [2].

In the past, transmission of the virus has primarily occurred from animals to humans, with only limited instances of human-to-human spread, typically involving short chains of transmission [3]. The virus can be transmitted through various routes, including respiratory droplets, body fluids, secretions, wounds and contact with contaminated materials [4]. In current outbreaks, a high proportion of cases was linked to sexual transmission, particularly among men who have sex with men (MSM), shifting the disease’s character from a zoonosis to a sexually transmitted infection [5].

The clinical course of mpox appears to be mild in the majority of cases [6]. The initial phase of infection often presents with prodromal symptoms and nonspecific manifestations of illness. As the disease progresses, localized to disseminated skin lesions can occur, characterized by a progression from macules to papules, vesicles, pustules, and finally crusts. These skin manifestations, depending on the mode of transmission, most commonly affect the anogenital region and the face/perioral area. Additionally, mucosal involvement in the rectal, pharyngeal, and urethral areas can occur. Mpox infection is typically self-limiting, with a duration of approximately 14 to 21 days [7]. However, according to older data from Africa, 1–3% of cases can lead to severe and sometimes fatal outcomes [8].

Severe cases may cause significant skin and mucosal involvement, ulceration, and scarring. However, limited data is available on the long-term consequences of MPXV infections, particularly regarding dermatological aspects. Therefore, this study aims to investigate the clinical spectrum of mpox, its impact on quality of life (QoL), sexuality and scarring during follow-up, in order to better understand the disease and its potential long-term effects on affected individuals.

To address these knowledge gaps, we conducted a prospective cohort study to assess disease characteristics during the initial infection and to investigate sequelae at 4–6 months of follow-up.

## Methods

### Study design and ethics

The present prospective cohort study of patients diagnosed with mpox was approved by the local ethics committee (application number EA2/139/22) and all patients provided written informed consent prior to their participation.

### Setting and eligibility

Adult patients diagnosed with MPXV infection by PCR testing of lesion swabs at Charité Universitätsmedizin Berlin treated at the Department of Dermatology and the Department of Infectious Diseases between May and September 2022 were consecutively enrolled. All participants received treatment according to standard clinical protocols, including, as indicated, topical antiseptics, systemic antibiotic treatment, analgesia and supportive care. All patients were offered testing for HIV and syphilis. Six of the cases included in this study have been previously reported in a case series during the onset of the mpox epidemic by some of the investigators [9]. Additionally, for some of these patients a plasma proteome analysis has been conducted and published [10].

### Variables

At baseline, the clinical and patient characteristics were assessed by the treating physicians using standardized questionnaires on demographic data, sexual behavior, vaccination status, comorbidities, concomitant medication, symptoms and treatment. Included was an assessment of pain and itching on a numeric rating scale (NRS) from 0 indicating no pain or itch to 10 indicating the most severe pain or itch, respectively.

Participants were invited to a follow-up visit 4 to 6 months after the initial diagnosis to assess scarring and QoL. The assessment of scars in this study was conducted using the Patient and Observer Scar Assessment Scale (POSAS), a validated tool for evaluating the appearance and quality of scars. The POSAS consists of two components: the patient scale (PSAS) and the observer evaluations (OSAS) of a scar [11]. Generally, a higher POSAS sum score indicates poorer scar quality, while a lower score indicates a better-quality scar [11]. While most mpox patients present with multiple lesions, the POSAS scale is designed for evaluating individual scars. To address this and generate meaningful and comparable data, we instructed patients to assess the scar that caused them most concern and study personnel to evaluate the most prominent scar.

The impact of mpox on patients’ QoL was evaluated using the Dermatology Life Quality Index (DLQI), a widely used questionnaire in dermatology [12]. The DLQI consists of 10 questions that address various aspects of daily life. The results of the DLQI range from 0 to 30 points, with a score of 0–1 indicating no impact on quality of life (QoL), 2–5 indicating a minor effect on QoL, 6–10 indicating a moderate effect on QoL, 11–20 indicating a major effect on QoL, and 21–30 indicating a severe effect on QoL [12].

Furthermore, the participants’ overall impairment, sexual impairment, and pain levels attributed to the mpox sequelae during the two weeks prior to the follow-up visit were assessed on a NRS ranging from 0 (indicating no impairment or pain) to 10 (indicating the most severe impairment or pain).

### Data collection

Pseudonymised study codes were used to assign the collected data to respective patients. During the acute infection, patients completed paper-based questionnaires, and the information was later entered into the electronic database by the study personnel. For the follow-up period, data collection was entered into the online database directly. Patients were provided with a link and a QR code to independently enter follow-up data on the DLQI and PSAS online. If questionnaires were not completed during the acute infection period, patients were able to provide the missing information later in the study by using links and QR codes that were sent to them.

### Data analysis

Descriptive statistics were used to determine the patient and clinical characteristics during the initial infection and follow-up visits. These included frequencies and proportions for categorical variables and medians with quartiles (Q1, Q3) for continuous variables. Mann-Whitney U tests were used to compare quality of life, sexual impairment, and scar quality 4–6 months after infection depending on bacterial superinfections or abscesses during the acute phase.

Statistical analyses were performed with SPSS Software version 27.0 (IBM Corp., Armonk, NY, USA).

## Results

A total of 43 patients were enrolled in the study, with a median age of 32.0 years and an age range of 19 to 64 years. Two participants were women (4.7%) and 41 (95.3%) were men, all of whom self-identified as MSM. Among the patients, 10 (23.3%) were HIV-positive and under effective antiretroviral therapy, while 33 (76.7%) were HIV-negative. Of the HIV-negative patients, 23 (69.7%) were using HIV pre-exposure prophylaxis (PrEP). One patient suffered from a chronic lung disease and one from diabetes. Two patients were iatrogenically immunosuppressed due to multiple sclerosis and psoriasis, receiving fingolimod and an anti-IL-17 antibody, respectively.

Only four patients (9.3%) were vaccinated against smallpox in their childhood, and none had recently been vaccinated against MPXV. It is worth noting that the modified vaccinia virus vaccination against mpox was not available in Germany until July 2022 [13].

The incubation period from the suspected event of infection until the onset of general symptoms was 7.5 days (median, Q1-Q3: 6.8–14.3). The median incubation period until the appearance of the first skin lesions was 9.0 days (Q1-Q3: 6.0–15.0). The median time between the first appearance of skin symptoms and a confirmed diagnosis by PCR was 5.0 days (Q1-Q3: 1.5–10.0). If hospitalization was necessary, the median duration of the hospital stay was 5.0 days (Q1-Q3: 4.0–6.0). Table 1 illustrates the epidemiological data on the course of the disease.

**Table 1:** Epidemiological data on the course of the disease

**Incubation period until onset of general symptoms (days), n = 18**		
Median (Q1– Q3)	7.5	(6.8–14.3)
Min– Max	2–35	
**Incubation period until appearance of first skin lesions (days), n = 21**		
Median (Q1– Q3)	9.0	(6.0–15.0)
Min– Max	2–35	
**Duration from appearance of skin lesions to molecular diagnosis (days), n = 25**		
Median (Q1– Q3)	5.0	(1.5–10.0)
Min– Max	-12–47	
**Length of hospital stay (days), n = 20**		
Median (Q1– Q3)	5.0	(4.0–6.0)
Min– Max	2–13	

### Characteristics of acute infection

The most common clinical symptoms during the acute infection were visible skin or mucosal lesions (40 out of 43 patients, 93.0%), lymphadenopathy (26/30, 86.6%) and fever (21/30, 70.0%). Overall, the patients presented with a median number of 6.5 skin lesions (Q1-Q3: 3.3–10.0, range: 0-200), although one patient under fingolimod treatment had 200 lesions [14].

Among the 30 patients who responded to these questions, pain was reported by 22 (73.3%), with a median pain NRS score of 8 (Q1-Q3: 6.0–10.0). Itching was observed in 20 patients (20/29, 69.0%), with a median itch NRS score of 6.5 (Q1-Q3: 4.3-8.0). Table 2 provides an overview of clinical characteristics during the acute infection.

**Table 2:** Clinical characteristics during the acute infection

		n	(%)
**Localization of lesions**^**1**^**(*n*** **= 35)**			
Genital		17	(48.6)
Anal		13	(37.1)
Mouth / Throat		9	(25.7)
Face / Neck		11	(31.4)
Torso		11	(31.4)
Limbs		13	(37.1)
**Pain, numerical scale from 0–10** (***n = 30)***			
Pain present		**22**	**(73.3)**
Median (Q1-Q3)	8.0 (6.0–10.0)		
Min-Max	2–10		
**Itching, numerical scale from 0–10** (***n = 30)***			
Itching present		**20**	**(69.0)**
Median (Q1-Q3)	6.5 (4.3-8.0)		
Min-Max	2–10		

^1^Multiple answers possible

Compared to genital localization, lesions in the anal area and in the mouth or throat were associated with a significantly higher proportion of pain (RR 3.60, 95%-CI 1.48–8.74; RR 2.83, 95%-CI 1.07–7.50, respectively). Itching was reported significantly more frequently only in the anal area (RR 2.94, 95%-CI 1.16–7.46), but not in the throat or mouth (Table 3).

**Table 3:** Pain and itching during acute phase according to the localization of lesions

Localization of lesions	*N*	With pain		Risk ratio (95%-CI)	With itching		Risk ratio (95%-CI), p^1^
		n	(%)		*n*	(%)	
Genital	17	4	(23.5)	*Reference*	4	(23.5)	*Reference*
Anal	13	11	(84.6)	3.60 (1.48–8.74), ***p*** **= 0.001**	9	(69.2)	2.94 (1.16–7.46), ***p*** **= 0.018**
Mouth / Throat	9	6	(66.7)	2.83 (1.07–7.50), ***p*** **= 0.047**	2	(22.2)	0.94 (0.21–4.20), *p* = 0.964
Face / Neck	11	3	(27.3)	1.16 (0.32–4.21), *p* = 0.831	2	(18.2)	0.77 (0.17–3.53), *p* = 0.775
Torso	11	3	(27.3)	1.16 (0.32–4.21), *p* = 0.831	3	(27.3)	1.16 (0.32–4.21), *p* = 0.831
Extremities	13	3	(23.1)	0.98 (0.26–3.64), *p* = 0.986	5	(38.5)	1.63 (0.54–4.90), *p* = 0.413

^1^Obtained from Mid-P exact test

Complications were reported in 20 (46.5%) patients (see Table 4), with 15 cases of either superinfection and/or the formation of abscesses. Of the 43 patients included in our cohort, 20 (46.5%) required hospitalization, mostly due to pain (*n* = 20) and/or superinfection (*n* = 10) or abscesses (*n* = 5). No fatalities were reported in this study.

**Table 4:** Complications upon diagnosis

Complications at diagnosis^1^ (*n* = 43)		
Patients with complications	**20**	**(46.5)**
- Bacterial superinfection (without abscess)	10	(50.0)
- Abscess	5	(25.0)
- Anal fissure	2	(10.0)
- Anal bleeding	1	(5.0)
- Epididymitis	1	(5.0)
- Epiglottitis	1	(5.0)
- Gastroenteritis	1	(5.0)
- EBV reactivation	1	(5.0)
- Infection-associated rash	1	(5.0)

^1^Multiple answers possible

Although not statistically significant, a notable association (RR 1.78, 95%-CI 0.99–3.19, *p* = 0.109) was observed between the HIV status of patients and the occurrence of complications (Table 5). Among the ten patients with known HIV infection, 70.0% (7/10) experienced complications, while only 39.4% (13/33) of the HIV-negative patients were affected. It is noteworthy that we did not detect any previously unknown HIV infections and that all patients with known HIV infections were receiving appropriate and effective antiretroviral therapy.

**Table 5:** Association between HIV status and complications

HIV status	*N*	Complications		No Complications		Risk ratio (95%-CI), p^1^
		*n*	(%)	*n*	(%)	
Total sample	43	20	(46.5)	23	(53.5)	
HIV-negative	33	13	(39.4)	20	(60.6)	*Reference*
HIV-positive	10	7	(70.0)	3	(30.0)	1.78 (0.99–3.19), *p* = 0.109

^1^Obtained from Mid-P exact test

The patients were provided with best supportive care as part of their treatment. Among the therapeutic measures administered, local antiseptics were used in 84.2% (32/38) of cases, while systemic antibiotic treatment was given to 65.9% (27/41) of patients. Analgesic medication was administered to all individuals, with 13.9% (5 out of 36) requiring intravenous administration. Access to tecovirimat, an mpox-specific antiviral drug, was severely limited in Germany in summer 2022. As none of the patients was critically ill, tecovirimat was not administered.

### Sequelae at 4-6-month follow-up

Nineteen of 43 patients (44.2%) were available for the follow-up visits at 4 to 6 months after the acute infection. Of these, 16 patients (84.2%) had residual skin lesions. Scarring was generally rated to be minimal by both patients (PSAS median: 14.0, Q1-Q3: 9.8–27.0) and observers (OSAS median: 13.5, Q1-Q3: 9.3–19.8). While 12 out of 19 patients (63.2%) reported no residual impairment due to their mpox disease at all, 2 out of 17 patients (11.8%) reported still having pain. Considering the overall sample, the DLQI was found to be low, with a median score of 1.0 (Q1-Q3: 0-2.5) (Table 6), indicating no significant impact on overall QoL. Likewise, general impairment (median: 0.0, Q1-Q3: 0–2), sexual impairment (median: 0.0, Q1-Q3: 0–5) and pain (median: 0.0, Q1-Q3: 0–0) were low (Table 6). Six patients (35.3%) reported a DLQI score of 0.0, and 10 patients (52.6%) experienced no sexual impairment at all.

However, comparing patients who had an abscess or bacterial superinfection during the acute phase with patients who did not experience such complications, revealed significant differences: Patients with bacterial superinfection or abscess during the acute phase had more intense scarring than those without these complications (median PSAS 24.0 vs. 11.0, *p* = 0.039; median OSAS 18.5 vs. 11.0, *p* = 0.059). Accordingly, these patients also suffered from significantly greater limitations in their quality of life and sexuality (median DLQI: 2.0 vs. 0.0, *p* = 0.036; median sexual impairment NRS: 5.0 vs. 0.0, *p* = 0.017) (Table 6).

**Table 6:** Quality of life, sexual impairment and scar quality 4–6 months after infection, in relation to bacterial superinfections or abscesses during the acute phase

	Entire sample		Bacterial superinfection /abscess during acute infection		No bacterial superinfection/ abscess		*p* ^1^
**Dermatology Life Quality Index (DLQI)**							
N	17		9		8		
Median (Q1– Q3)	1.0	(0–2.5)	2.0	(1.0–5.5)	0.0	(0.0–1.0)	**0.036**
Min– Max	0–13		0–13		0–8		
**General impairment within the last two weeks (scale 0–10)**							
N	19		9		10		
Median (Q1– Q3)	0.0	(0–2)	1.0	(0.0–4.0)	0.0	(0.0–0.5)	0.143
Min– Max	0–6		0–6		0–5		
**Sexual impairment within the last two weeks (scale 0–10)**							
N	19		9		10		
Median (Q1– Q3)	0.0	(0–5)	5.0	(0.5–7.5)	0.0	(0.0–0.8)	**0.017**
Min– Max	0–10		0–10		0–7		
**Pain within the last two weeks (scale 0–10)**							
N	17		9		8		
Median (Q1– Q3)	0.0	(0–0)	0.0	(0.0–0.0)	0.0	(0.0–0.0)	0.864
Min– Max	0–3		0–1		0–3		
**Visible scarring (*n*** **= 19)**							
Scars present	16	(84.2)	8	(88.9)	8	(80.0)	> 0.999^2^
**Observer Scar Assessment Scale (OSAS)**							
N	16		8		8		
Median (Q1– Q3)	13.5	(9.3–19.8)	18.5	(12.3–22.8)	11.0	(9.0–13.8)	0.059
Min– Max	7–26		7–26		8–19		
**Patient Scar Assessment Scale (PSAS)**							
N	14		7		7		
Median (Q1– Q3)	14.0	(9.8–27)	24.0	(14.0–34.0)	11.0	(9.0–14.0)	**0.039**
Min– Max	6–45		10–45		6–27		

^1^Obtained from Mann-Whitney U test for continuous variables and from Fisher’s exact test for categorical variables

## Discussion

In this article, we present the findings of our prospective cohort study, which provide insight into the clinical spectrum of MPXV infections, including localization, symptoms, and complications during the acute phase. Furthermore, we report on the generally mild long-term consequences of the disease, while scar formation and the impact on patients’ quality of life and sexuality is found to be remarkable for the proportion of patients that suffered from complications during the acute infection. Our study results contribute to the current understanding of MPXV infections and to the development of better strategies for prevention, treatment, and management of this emerging disease. The limited number of publications on mpox sequelae highlights the need for further research in this area.

The clinical manifestations and locations of lesions observed in our study of patients with PCR-confirmed mpox are consistent with the results of a meta-analysis conducted in 2023, which combined published data up until September 2022 [6]. In a Belgian follow-up study examining sequelae at 7 to 20 weeks, 41.2% of patients reported impairment [15], which is in accordance with the quota of more than one third of patients reporting at least some impairment in our study after 4–6 months.

Patients with superinfections and abscesses during the initial infection were found to have a significantly worse long-term outcome. Therefore, efforts should be made to prevent and minimize these complications. The potential need for surgery and the resulting scarring due to surgical interventions should also be considered in the management of mpox patients.

### Limitations

However, this study encountered some limitations. While the study’s findings provide valuable insights, the generalizability of the results may be constrained due to the inclusion of a relatively strongly affected group of patients who have been treated at an university hospital. While in general, only a minority of patients required inpatient treatment [16, 17], our cohort showed a higher proportion with almost half of the cases requiring hospitalization.

Also, the study population was limited to patients with laboratory confirmed MPX virus infections, which may not reflect the clinical spectrum of the disease in the general population. It can be assumed that during waves of incidence in diseases with a heterogeneous symptom profile, a significant number of patients go through the illness without being diagnosed.

Additionally, the sample size was relatively small, with 43 individuals included at the time of diagnosis and 19 patients completing the follow-up period. We placed significant effort in contacting patients and motivating them to attend their follow-up visits; however, many patients reported no lingering effects and expressed a lack of interest in returning to the hospital for a dermatological assessment. Moreover, there was a considerable decrease in the mpox incidence rates in Germany towards the end of summer 2022, accompanied by a decline in media attention.

Considering the limitations, caution should be exercised when interpreting and extrapolating the results of this study. Future research with larger and more diverse cohorts, encompassing various healthcare settings, is warranted to further validate and expand upon these initial findings.

### Areas for future research

In cases of superinfection, the choice of antibiotics is a crucial consideration. Future studies could focus on evaluating the bacterial strains commonly found in superinfected lesions to guide appropriate antibiotic selection and optimize treatment outcomes.

The interplay between HIV-induced immunosuppression and the immune response to mpox infection could potentially influence the severity of disease manifestations, treatment outcomes, and long-term sequelae. Hence, the correlation between HIV status and complications is an important aspect to explore. Although the association between the HIV status of patients (all under effective antiretroviral therapy) and the occurrence of complications did not reach statistical significance, a notable trend was observed in our study (Table 5). In a recent systematic review of 99 published reports, the odds ratio was found to be significantly higher for skin rash, proctitis and diarrhea in people living with HIV during an mpox infection [18]. Future studies could investigate whether specific antiretroviral regimens and to what extent the immune status contributes to the observed association between HIV and mpox.

Psychological and social factors, including the impact of stigma and sexual impairment, should also be addressed in further research, as they may have longer-term effects on the well-being of mpox patients.

### Emerging incidence and future relevance

Starting in May 2023, the Centers for Disease Control and Prevention (CDC) and the Journal of the American Medical Association (JAMA Network) expressed concerns about the global resurgence of mpox cases, indicating the increasing relevance of this topic in the near future [19]. Despite a previous decline in cases following the peak in August 2022, recent data reveal a resurgence of mpox cases, particularly among individuals who were previously vaccinated [20]. This unexpected finding raises significant questions regarding the long-term effectiveness of vaccination and the potential waning of immunity over time. Consistent with recommendations from public health experts to maintain vigilance for new mpox cases, it is essential to consider proactive measures, such as strengthening prevention efforts and evaluating vaccine effectiveness, to effectively address this emerging challenge. Our study contributes to the growing body of evidence on the dynamic nature of mpox and emphasizes the importance of continuous research and vigilance in managing and controlling this infectious disease. Emphasizing the prevention of complications and timely initiation of antiseptic and/or antibiotic treatment may be crucial for optimizing the long-term outcomes of patients. The findings of our study provide actionable results that can be promptly implemented in response to emerging incidence waves.

## Conclusions

This prospective cohort study provides important insights into the clinical spectrum, QoL and scarring in patients with MPX virus infections.

A wide range of skin and mucosal involvement including visible or sensitive areas was observed in this sample of relatively severely affected mpox patients. After 4 to 6 months, the majority had inconspicuous scars, and their QoL was not significantly affected. However, patients with superinfection or abscess during the acute phase had significantly more extensive scar formation and experienced a significantly greater impairment of their QoL and sexuality. Taking these findings into consideration, it appears to be crucial to prevent such complications by providing adequate antiseptic and/or antibiotic treatment during the acute phase.

Further studies with larger sample sizes and longer follow-up periods are needed to confirm these findings and to determine the extent to which the observed sequelae are attributable to MPX virus infections. These efforts will contribute to a better understanding and management of mpox, especially in the context of its rising incidence in certain regions of the world.

## Data Availability

No datasets were generated or analysed during the current study.

## Abbreviations

MPXV: mpox virus
OSAS: Observer Scar Assessment Scale
PCR: Polymerase Chain Reaction
POSAS: Patient and Observer Scar Assessment Scale
PSAS: Patient Scar Assessment Scale
QoL: quality of life
